# Super-Protective Child-Rearing by Japanese Bess Beetles, *Cylindrocaulus patalis*: Adults Provide Their Larvae with Chewed and Predigested Wood

**DOI:** 10.3390/insects7020018

**Published:** 2016-04-26

**Authors:** Tatsuya Mishima, Noriko Wada, Ryûtarô Iwata, Hirosi Anzai, Tadatsugu Hosoya, Kunio Araya

**Affiliations:** 1 Biosystematics Laboratory, Graduate School of Social and Cultural Studies, Kyushu University, Fukuoka 819-0395, Japan; araya@scs.kyushu-u.ac.jp; 2 Laboratory of Biotechnology in Daily Life, Department of Bioscience in Daily Life, Nihon University, Fujisawa, Kanagawa 252-0880, Japan; wada.noriko@nihon-u.ac.jp (N.W.); anzai.hiroshi@nihon-u.ac.jp (H.A.); 3 Laboratory of Forest Zoology, Department of Forest Science and Resources, College of Bioresource Sciences, Nihon University, Fujisawa, Kanagawa 252-0880, Japan; iwata@brs.nihon-u.ac.jp; 4 Institute of Decision Science for a Sustainable Society, Kyushu University, Fukuoka 819-0395, Japan; tadatsugu.hosoya.848@m.kyushu-u.ac.jp

**Keywords:** super-protective child-rearing, bess beetles, *Cylindrocaulus patalis*, rotten wood, glycosidase activity, glycanase activity, mycophagy, chewed and predigested wood by the parents

## Abstract

Beetles of the family Passalidae (Coleoptera: Scarabaeoidea) are termed subsocial. The insects inhabit rotten wood as family groups consisting of the parents and their offspring. The Japanese species *Cylindrocaulus patalis* has the lowest fecundity among passalids because siblicide occurs among the first-instar larvae; accordingly, parental care toward the survived larva is the highest among Passalidae. To clarify the nutritional relationships between the parents and their offspring, we investigated their ability to digest three types of polysaccharides that are components of wood (cellulose and β-1,4-xylan) and fungal cell walls (β-1,3-glucan). Although carboxymethyl-cellulase activity was barely detectable, β-xylosidase, β-glucosidase, β-1,4-xylanase and β-1,3-glucanase activities were clearly detected in both adults and larvae. Because the activities of enzymes that digest β-1,3-glucan were much higher than those for degrading β-1,4-xylan, in both adults and larvae, it is concluded that they are mainly fungivorous. Furthermore, these digestive enzymatic activities in second- and third-instar larvae were much lower than they were in adults. Although all larval instars grew rapidly when fed chewed wood by their parents, larvae ceased growing and died when fed only artificially ground wood meals. We conclude that the larvae are assumed to be provided with chewed predigested wood in which β-1,3-glucan is degraded by parental enzymes.

## 1. Introduction

The major components of wood, accounting for about 90% of its mass, are cellulose, lignin and hemicelluloses [1], compounds that are difficult to digest by many organisms. Wood is also a poor food resource because it possesses extremely low nitrogen content with C/N ratios in the range of 250 to 1250 [2,3]. Wood feeders must therefore overcome the difficulties of digesting wood cell-wall components and of obtaining supplemental nitrogen. Many wood-rot fungi are capable of digesting cellulose, hemicellulose and lignin to some degree and are also capable of concentrating their nitrogen contents. Consequently, rotten wood is a better food resource than dead wood [4]. Many species of larval scarabaeoid Coleoptera (Passalidae, Lucanidae, Glaphyidae, Ceratocanthidae, Hybosoridae and many subfamilies of Scarabaeidae) inhabit and utilize rotten wood (white rotten wood, brown rotten wood and soft rotten wood) [5,6]. In these non-social species, the larval and adult feeding habits differ, with the larval stages generally utilizing rotten wood or humus. In contrast, in some subsocial species (e.g., Figulinae of Lucanidae and Passalidae; Scarabaeoidea) the larvae live together with their parent(s) in rotten wood [7]. The drivers for the evolution of subsocial species are thought to reflect the qualitative and quantitative limitations of wood and rotten wood as foods [7,8,9,10] and the properties of narrow spaces in rotten wood that form tough, protective shelters for the family group [7,11].

In many scarabaeoid species, parental care is critical for the proper growth and development of their offspring [12,13,14]; provision of an energy-rich, high-quality diet improves their growth rate and reduces the duration of development, increasing survival [12,15,16,17,18,19,20,21,22,23,24]. For many wood-feeding arthropods, nutritional supplementation by parents is essential to the growth and development of the progeny [25].

The level of parental care shown by Passalidae (so-called bess beetles) is considered to be the highest among subsocial insects inhabiting rotten wood [26,27]. Many species of Passalidae live as family groups (male and female mature parents, eggs, larvae, pupae, and newly emerged immature and mature adults) within rotten wood [28]. Four types of parental care have been reported in Passalidae: (1) care and protection of the eggs; (2) excavation of galleries; (3) feeding of larvae; and (4) pupal cell building [29,30,31,32,33,34,35]. Immature and newly emerged adults play a “helper” role for the reproducing adults in the care of siblings [11,28]. With regard to feeding larvae (3), in passalids, mature and immature newly emerged adults provide sibling larvae with chewed wood and feces, which chemical analysis has shown contains a higher nitrogen concentration than ingested wood [28,34,36,37,38]. Typically, all of these stages (mature parents, larvae, and mature and immature newly emerged adults) consume the feces of the mature parents. It is anticipated that the feces are composed of wood that has been fragmented, digested, and inoculated with bacteria and fungi from the digestive tract of the mature parents [28]. The proposed advantages of feeding on feces by all stages are: (1) effective provision of an organ of external digestion; (2) provisioning the offspring with symbiotic microorganisms that are able to digest cellulose and lignin; and (3) recycling of the nitrogen source [7,11].

Although the larvae of many species of lucanids are able to live in rotten wood without parental assistance/care, the total duration of their larval development is reported to be two years or more [7,39,40,41]. In contrast, because of the brood care exhibited by Passalidae, the period of their larval development is the shortest (at most several months) among insects inhabiting rotten wood [7,10,11,26,28]. Indeed, the larval period of *Cylindrocaulus patalis* (subfamily: Aulacocyclinae) is estimated to be about one month [42], which is extremely short even among the Passalidae [7,10,11,43,44]. This character may reflect the fecundity, siblicide and elaborate parental care exhibited by this species. Indeed, the fecundity of *C. patalis*, which produces only one or two eggs, is the lowest recorded among passalids [7,42]. After hatching, siblicide occurs among first-instar larvae and a single larva survives [42,45]. Although both male and female parents provide larvae of all instars with chewed wood and parental feces, the larvae are usually indifferent to feces presented directly [42]. Therefore, it is thought that it is essential for the larva of all stages to feed on chewed wood. Feeding of larvae with chewed wood occurs in two ways: either directly between the parental and larval mouthparts (“mouth-to-mouth feeding”) or, occasionally, larvae consume chewed wood that is dropped on the substrate by the parents (“drop-and-pickup feeding”) [42]. In addition, the female parent provides the third (final)-instar larva with trophic eggs as food; this food is not supplied to first- and second-instar larvae [46]. *C. patalis* has the northernmost distribution of the passalids, inhabiting mountainous areas (>1000 m altitude) in Shikoku and Kyushu, southwest Japan [42,43,44,47], located in the cool temperate zone [42]. The larvae must complete their development during the summer period and they fail to overwinter and die if they do not become adults before the winter [44]. The parental care exhibited by this species is believed to contribute to the shortening of the larval period. Larvae of *C. patalis* are unable to chew rotten wood and they cease growing and die if isolated and fed with only artificially ground wood meals [7]. However, they grow very rapidly if fed chewed wood processed by their parents in the field [7,42]. Clearly, the larvae are extremely dependent on their parents [7].

The nutritional relationships between the parents and larvae of Passalidae have been the focus of a number of studies of their nutritional ecology and ethology [27,31,34,38,42,45,46,48,49,50,51,52], and of their nutritional physiology with respect to wood decomposition and microbiology [53,54,55,56,57,58,59]. However, few studies have dealt with biochemical and nutritional aspects of passalid larval ingestion of chewed wood or feces produced by the parents.

To further elucidate the food habits of the adult and larva, and the nutritional relationships between the parents and larvae of Passalidae, we carried out a biochemical study of *C. patalis*, the species reported to exhibit the highest level of brood care among insects inhabiting rotten wood. We focused on the abilities of both larvae and adults to digest three types of polysaccharides that are main components of wood (cellulose and β-1,4-xylan) and fungal cell walls (β-1,3-glucan) and compared their activities for saccharification of polysaccharides.

## 2. Materials and Methods

### 2.1. Insects

**Figure 1:**
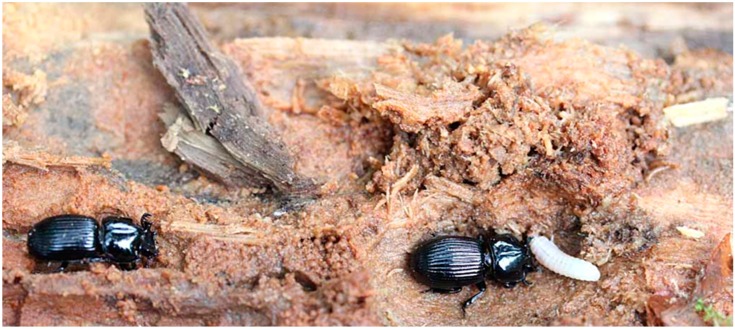
The rotten wood habitat of adult beetles *Cylindrocaulus patalis* and their larval offspring in the field.

Adults (*n* = 9) and larvae (second instar, *n* = 2; third instar, *n* = 3) of *C. patalis* (Figure 1) were collected at Mount Kunimi, Yatsushiro, Kumamoto Prefecture, Japan on 9 July 2013.

First-instar larvae were not used in this study because their body sizes were too small to obtain an adequate quantity of crude enzyme solution for the assays.

### 2.2. Chemicals

The substrates used for the assays of glycosidase and glycanase activities were cellulose and β-1,4-xylan (wood cell wall components) and β-1,3-glucan (fungal cell wall component). The substrates of the glycosidase used in this study, *p*-nitrophenyl (PNP)-β-d-glucoside and PNP-β-d-xyloside, were purchased from Sigma-Aldrich, St. Louis, MO, USA. A 10 mM solution of each substrate was used. Sodium carboxymethyl cellulose (CMC; Cellogen^®^ BS) was purchased from Dai-ichi Kogyo Seiyaku, Kyoto, Japan. The β-1,4-xylan (derived from oat spelts) was purchased from Sigma-Aldrich, and β-1,3-glucan (curdlan) was purchased from Wako Pure Chemical Industries, Osaka, Japan. The β-1,4-xylan and β-1,3-glucan were reduced by NaBH_4_. After preparing the carbohydrate solutions and suspensions, the concentrations were measured using the phenol-sulfuric acid method [60] and adjusted to 0.71% (*w*/*v*) in terms of the concentration of the main constituent monomer of each sugar.

*N*-(2-Acetamido)-2-aminoethanesulfonic acid (ACES) and Coomassie Brilliant Blue-G250 were purchased from Sigma-Aldrich, while tris (hydroxymethyl) aminomethane (Tris), 3,3-dimethylglutaric acid (DGA), 2-amino-2-methyl-1,3-propanediol (AMP) and bovine serum albumin were purchased from Wako Pure Chemical Industries.

ACES buffer (100 mM, pH 7.5) containing 500 mM NaCl was used for preparing gut extracts. A 300-mM “GTA” buffer, consisting of 100 mM DGA, 100 mM Tris, and 100 mM AMP, was used as the reaction buffers.

GTA buffer solutions used for each of the substrates followed Wada *et al.* [61]. They consist of CMC and β-1,4-xylan, GTA buffer solution (pH 7), and β-1,3-glucan, PNP-β-d-glucoside and PNP-β-d-xyloside, GTA buffer solution (pH 5).

### 2.3. Extract of Crude Enzyme Solution and Protein Concentration

After collecting *C. patalis*, the adults and their own larvae were reared in decayed wood fragments for three days. Then, the body surfaces of each insect were sterilized with 70% ethanol and they were placed in individual Falcon tubes (50 mL tube for the adults and 1.5 mL tubes for the second- and third-instar larvae). The buffer solution used to extract crude enzyme solution was 100 mM ACES buffer (pH 7.5) containing 500 mM NaCl [61,62]. The samples were homogenized with a glass rod (for the adults) or a homogenizer pestle (for the larvae) and suspended in ACES/NaCl solution (pH 7.5). The suspensions were centrifuged for 15 min at 10,000× *g* at 4 °C and the supernatants were used for the assays of enzymatic activity. Protein concentrations were determined as described by Bradford [63].

### 2.4. Glycosidase Assays

A 200 μL volume of reacting fluid containing 10 mM PNP-glycoside, 300 mM GTA buffer and 50 μL of crude enzyme solution was incubated at 30 °C. The reaction was stopped by the addition of 4 mL of 100 mM sodium carbonate solution and PNP was quantified based on the absorbance at 420 nm. One unit of glycosidase activity (1 U = 1000 mU) was defined as the activity needed to produce one μmole PNP per minute [62].

### 2.5. Glycanase Assays

A 50 μL volume of each extract was mixed with 700 μL of 0.71% substrate solution and 250 μL of 300 mM GTA buffer and incubated at 30 °C. The concentrations of reducing sugars were determined using the Somogyi-Nelson method [64,65] modified by Anzai *et al.* [62,66]. Briefly, the reaction mixtures were mixed with 1000 μL of Somogyi-Nelson copper reagent, boiled for 15 min, chilled quickly on ice and then mixed with 1000 μL of Nelson reagent. After 15 min, the mixtures were diluted with 3000 μL of distilled water and their absorbances at 500 nm were measured using a spectrophotometer (UV1600; Shimadzu, Kyoto, Japan). One unit of glycanase activity (1 U = 1000 mU) was defined as the activity needed to produce reducing sugars equivalent to 1 μmole of monomeric sugar from the substrate polysaccharide per minute.

### 2.6. Data Analysis

Because of the paucity of larvae, we did not perform statistical comparisons between the different instars.

## 3. Results

### 3.1. Glycosidase Activity

Glycosidase activities of the adults and larvae are shown in Table 1.

In the adults, and second-instar and third-instar larvae, both β-glucosidase and β-xylosidase activities were detected.

Total activities of β-glucosidase were approximately 23 times, 157 times and 14 times as high as the β-xylosidase activities in adults, second-instar larvae and third-instar larvae, respectively. Likewise, the specific activities (per mg protein) of β-glucosidase were approximately 24 times, 100 times and 12 times as high as the respective β-xylosidase activities, while the specific activities (per g body weight) of β-glucosidase were approximately 23 times, 122 times and 14 times as high as the corresponding β-xylosidase activities.

Comparing glycosidase activities among the adults and larvae, the total activities of β-glucosidase and β-xylosidase were markedly higher in adults than in the second- and third-instar larvae. Specific activities (per mg protein and per g body weight) of β-glucosidase in second-instar larvae were approximately 19 times and three times as high as those in third-instar larvae, respectively, although the total activities of β-glucosidase in second- and third-instar larvae were similar. The specific activity (per mg protein) of β-xylosidase in second-instar larvae was approximately twice as high as that in third-instar larvae, although the total activity and specific activity (per g body weight) of β-xylosidase in third-instar larvae were approximately 15 times and three times as high as in second-instar larvae, respectively.

### 3.2. Glycanase Activity

The activities of the polysaccharide digestive enzyme are shown in Table 2.

Although carboxymethyl (CM)-cellulase, β-1,4-xylanase and β-1,3-glucanase activities were detected in the adults and second- and third-instar larvae, CM-cellulase activities were weakest in all stages.

In the adults, β-1,3-glucanase activities were approximately 12, 15 and 13 times as high as β-1,4-xylanase activities in terms of total activity (mU), specific activity (mU/mg protein), and specific activity (mU/g body weight), respectively. Likewise, in the second-instar larvae, the respective measures of β-1,3-glucanase activity were all approximately five times as high as β-1,4-xylanase activities, and in the third-instar larvae, they were approximately seven, seven and eight times as high.

Glycanase activities were compared among the three stages. Although specific activities (mU/mg protein and mU/g body weight) of β-1,4-xylanase in the adults were as high as those in second-instar larvae, the total activity of β-1,4-xylanase in adults was approximately four times as high as in second-instar larvae. The β-1,4-xylanase activity in adults was approximately 10, 52 and five times as high as in third-instar larvae in terms of total activity (mU), specific activity (mU/mg protein), and specific activity (mU/g body weight), respectively. Likewise, values of β-1,3-glucanase activity in adults were approximately 11, four and two times as high, respectively, as the corresponding measurements in second-instar larvae, and values of β-1,3-glucanase activity in adults were approximately 16, 105 and 9 times as high as those in the third-instar larvae.

The β-1,4-xylanase activity in second-instar larvae was approximately two, 45 and seven times as high as that in third-instar larvae in terms of total activity (mU), specific activity (mU/mg protein), and specific activity (mU/g body weight), respectively. Likewise, β-1,3-glucanase activity in the second-instar larvae was also approximately two, 30 and five times as high as that in the third-instar larvae, respectively.

## 4. Discussion

This study on the glycosidase and glycanase activities in three life stages of *C. patalis* indicated that enzymatic systems for degrading CMC, β-1,4-xylan and β-1,3-glucan are present in both the adults and larvae. However, because CM-cellulase activity was much lower than the other enzymatic activities in all samples, it is likely that the last two substrates represent the main food resource of *C. patalis*. Comparison of the glycosidase and glycanase activities in the degradation of β-1,4-xylan and β-1,3-glucan indicates that *C. patalis* utilizes much more β-1,3-glucan than β-1,4-xylan. Therefore, it is suggested that both the adults and larvae are mainly fungivorous.

Our study indicated that the digestive enzymatic activities for degrading β-1,3-glucan in the second- and third-instar larvae were much lower than those of their parents. The larval digestive enzyme system appears unable to hydrolyze and effectively utilize β-1,3-glucan. Therefore, the significance of parental care in *C. patalis* may be related to polysaccharide saccharification activity. In fact, the larvae of this species cease growing and die when isolated from their parents because they cannot chew rotten wood, or even utilize artificially ground wood meals [7]. However, the larvae grow very rapidly if fed chewed wood processed by their parents in the field [7,42]. This implies that the chewed wood includes nutrients or enzymes essential for the larvae. It was suggested that the parent insects directly provide their own larvae with digestive enzymes that help to digest wood [42]. Combining ethological information and the present results, it is possible that *C. patalis* parents provide their larvae with the digestive enzyme for degrading β-1,3-glucan through mouth-to-mouth feeding of chewed wood. However, the digestive enzymatic activities in second- and third-instar larvae were much lower than that in their parents and it is possible that enzymes derived from their parents are inactivated or decomposed by microorganisms before entering the larval gut. Therefore, it is likely that the larvae utilize chewed wood, including fungal cell walls, predigested by the parental enzymatic system for degrading β-1,3-glucan. The activities of all digestive enzymes in the second-instar larvae were also higher than those in third-instar larvae. Because the quantity of food ingested significantly affects the digestive enzymatic activity [67], we conclude that second-instar larvae actively feed on and utilize more chewed wood than third-instar larvae do. However, we suggest that third-instar larvae need not feed on chewed wood because the female parent provides only third-instar larva with trophic eggs, which they consume [46]. It is presumed that the nutritional value of the trophic eggs is much higher than that of wood and wood-rot fungi, which suggests that trophic eggs are an efficient food resource for the third-instar larva. The trophic egg may satisfy most of the nutrition requirements of the third-instar larvae [42,46]. Thus, it is highly possible that the provision of chewed wood by both parents and the trophic eggs produced by the female parent greatly are essential for their offspring’s survival and growth rate.

*C. patalis* has the northernmost distribution among passalids, inhabiting the mountainous area in the cool temperate zone [42,43,44,47]. The larvae fail to overwinter and die if they cannot become adults during the warm summer months [44]. These ecological, ethological, and physiological factors must influence the short larval period in this species. The very small clutch size of *C. patalis* permits maximum investment in rearing of a single larva and also must contribute to the extremely short larval period. Specifically, the parents provide chewed wood for all instars [42] and trophic eggs for only the third instar. The larvae do not possess suitable jaws for excavating and chewing rotten wood [42] but the adults prepare chewed wood, which is consumed by the larvae [42]. Therefore, larvae of all instars avoid consumption and expenditure of the large amounts of energy needed to generate wood chips. The glycosidase and glycanase activities in the second- and third-instar larvae suggest that they mainly digest and utilize wood-rot fungi. Wood-rot fungi probably contain a wide range of nutrients and the larvae also utilize β-1,3-glucan in the fungal cell wall, predigested by their parents, and supplied through mouth-to-mouth feeding. Utilization of wood-rot fungi by larvae of all instars probably contributes to the shortening of the larval period. Furthermore, the provision of the third-instar larvae with a nutritious trophic egg by its female parent must also help to accelerate larval development during the warm summer period. Instead, much energy is expended by the parents, living together with their larvae, in the production of chewed wood and trophic eggs. The parents need to digest the fungi as well as rotten wood to maintain these highly energetic activities.

Even in species of Passalidae with higher fecundity and relatively lower levels of brood care than that exhibited by *C. patalis*, some degree of siblicide occurs among the larvae, and similar rearing behavior is performed by adults, except for the production of trophic eggs [45,46]. The larval period of most passalid beetles, which is usually about several months, is much longer than that of *C. patalis* [7]. We propose that the larval periods of passalid beetles depend on their levels of brood care. Possibly, the level of brood care exhibited by most species is relatively lower than in *C. patalis* but the larval ability to digest rotten wood (particularly wood-rot fungi) is higher than in *C. patalis*. A similar correlation has been shown between the level of brood care and wood-digestion ability in the larvae of xylophagous cockroaches [68,69]. Therefore, it would be interesting to investigate polysaccharide saccharification activity in passalid genera in which the level of brood care is lower than in *C. patalis*, e.g., *Aceraius* and *Leptaulax*.

Larval growth rates are also affected by nitrogen content [70,71] but the rotten wood that passalid beetles inhabit usually contains very low levels of nitrogen (the C/N ratio of rotten wood usually ranges from 100 to 200 [2]. Many species of insects inhabiting dead and rotten wood are able to fix atmospheric nitrogen to supplement the nitrogen source, including termites [72,73,74,75], a bark beetle [76], a xylophagous cockroach [77], a scarabaeid beetle [78], a stag beetle [79] and a passalid beetle [80]. Whether adults of *C. patalis* fix atmospheric nitrogen should be investigated.

## 5. Conclusions

Digestive enzymatic activities for CMC, β-1,4-xylan and β-1,3-glucan were detected in the adults and second- and third-instar larvae of *Cylindrocaulus patalis*. In terms of biochemistry, the adults of this species are capable of feeding on and gaining nutrients from rotten wood. Because CM-cellulase activity was much weaker than the β-1,4-xylanase and β-1,3-glucanase activities, the main food of this species is β-1,4-xylan and β-1,3-glucan. The digestive enzymatic activity for degrading β-1,4-xylan is lower than for β-1,3-glucan. Therefore, it is considered that *C. patalis* mainly feeds on the mycelium of wood-rot fungi. The adults have a full enzymatic system for degrading β-1,3-glucan, whereas in larvae this system is incomplete. Therefore, the larvae are assumed to be provided by the parents with chewed wood predigested by digestive enzymes derived from parents. The carbohydrase activities in the third-instar larvae were lower than those in the second-instar larvae but trophic eggs provided by their female parent are able to satisfy most of the nutritional requirements of the third-instar larva. The apparently super-protective care by the adult is thought to facilitate the extreme brevity of the larval period.

## Figures and Tables

**Table 1:** Glycosidase activities in whole bodies of *Cylindrocaulus patalis*.

Sample	Substrate	Incubation Time (min)	Total Activity (mU)	Specific Activity	
				(mU/mg Protein)	(mU/g Whole Body)
Adults (*n* = 9)	PNP-β-glucoside	180	246.91 ^a^ ± 28.30 ^b^	491.56 ± 248.51	663.29 ± 55.49
	PNP-β-xyloside	180	10.59 ± 1.18	20.61 ± 10.36	28.77 ± 2.68
Second-instar larvae (*n* = 2)	PNP-β-glucoside	180	6.28 ± 0.57	39.08 ± 7.36	92.80± 2.26
	PNP-β-xyloside	180	0.04 ± 0.03	0.39 ± 0.28	0.76 ± 0.54
Third-instar larvae (*n* = 3)	PNP-β-glucoside	180	8.12 ± 0.91	2.06 ± 0.36	34.13 ± 2.72
	PNP-β-xyloside	180	0.59 ± 0.18	0.17 ± 0.08	2.40 ± 0.75

^a^ means; ^b^ SE, standard error; whole body weight of each sample is as follows: adult, 0.223–0.552 g; second-instar larvae, 0.057–0.079 g; third-instar larvae, 0.195–0.329 g.

**Table 2:** Glycanase activities in whole bodies of *Cylindrocaulus patalis*.

Sample	Substrate	Incubation Time (min)	Total Activity (mU)	Specific Activity	
				(mU/mg Protein)	(mU/g Whole Body)
Adults (*n* = 9)	CM-cellulose	180	0.50 ^a^ ± 0.47 ^b^	0.53 ± 0.50	1.60 ± 1.51
	β-1,4-xylan	180	52.25 ± 6.15	83.00 ± 35.26	133.78 ± 5.06
	β-1,3-glucan	180	639.37± 87.21	1205.99 ± 596.57	1695.16 ± 145.14
Second-instar larvae (*n* = 2)	CM-cellulose	180	3.12 ± 2.21	28.25 ± 19.97	54.80 ± 38.75
	β-1,4-xylan	180	12.48 ± 2.79	71.84 ± 3.73	178.80 ± 20.65
	β-1,3-glucan	180	58.70 ± 11.36	344.24 ± 29.23	847.20 ± 70.14
Third-instar larvae (*n* = 3)	CM-cellulose	180	3.06 ± 2.03	1.28 ± 0.98	14.27 ± 10.21
	β-1,4-xylan	180	5.37 ± 0.70	1.60 ± 0.53	24.55 ± 5.53
	β-1,3-glucan	180	39.16 ± 11.51	11.54 ± 3.84	186.89 ± 66.78

^a^ means; ^b^ SE, standard error; whole body weight of each sample is as follows: adult, 0.223–0.552 g; second-instar larvae, 0.057–0.079 g; third-instar larvae, 0.195–0.329 g.
